# Apospory Expressivity and Segregation in Intraspecific Hybrids of *Paspalum notatum* Flügge

**DOI:** 10.3390/genes17101220

**Published:** 2026-10-01

**Authors:** Andrea Peréz Góez, Carine Simioni, Diógenes Cecchin Silveira, Nair Dahmer, Maria Eduarda Pagani Sangineto, Jonathan Krug, Cristiane Stainhaus, Matheus Bragé dos Santos, Henrique Ludvig Vogt, Pablo Przyczynski Rauber, Roberto Luis Weiler

**Affiliations:** Department of Forage Plants and Agrometeorology, Federal University of Rio Grande do Sul (UFRGS), Porto Alegre 91501-970, RS, Brazil; andrea.perezg@udea.edu.co (A.P.G.); carine.simioni@ufrgs.br (C.S.);

**Keywords:** apospory, reproductive mode, cytoembryology, segregation, forage breeding, *Paspalum notatum*

## Abstract

**Background/Objectives:** Apospory is a key component of gametophytic apomixis in *Paspalum notatum* and is relevant to breeding because, together with parthenogenesis and appropriate endosperm development, it can enable clonal seed reproduction. This study determined reproductive phenotype at the embryo-sac level, quantified apospory expressivity, and evaluated segregation in tetraploid intraspecific hybrids. **Methods:** Inflorescences were collected from 84 hybrids from 11 crosses between sexual female and aposporous male parents. Cleared ovaries were examined by differential interference contrast microscopy. Hybrids were classified as facultatively aposporous when at least one ovule contained an aposporous embryo sac; expressivity was calculated as A/(S + A) × 100. **Results:** Reproductive phenotype was determined in 80 hybrids: 48 were facultatively aposporous and 32 were sexual; four were indeterminate. Expressivity ranged from 3% to 69%. Pooled segregation did not deviate significantly from 1:1 (χ^2^ = 3.20; *p* = 0.0736), whereas the largest family, KF4 × KF6, deviated from 1:1 (15 S:32 facultatively aposporous; exact *p* = 0.0186). Between-family heterogeneity was not significant (Monte Carlo exact *p* = 0.1444). **Conclusions:** Apospory showed substantial variation in expressivity and family-level segregation. Because parthenogenesis and maternal progeny origin were not evaluated, these results characterize apospory rather than complete apomictic seed reproduction.

## 1. Introduction

The natural grasslands of the Pampa biome are among the most important open ecosystems in South America, harboring high herbaceous diversity and supporting extensive livestock production [1]. These permanent grasslands also provide an important source of forage for ruminants. Consequently, increasing forage productivity while maintaining the ecological integrity of native grasslands is an important strategy for reconciling livestock production with biodiversity conservation [2].

The genus *Paspalum* L. is widely distributed across South American grasslands and includes several species of ecological and agronomic importance [3,4]. Among them, *Paspalum notatum* Flügge is a perennial, rhizomatous forage grass adapted to a broad range of environmental conditions. Native biotypes and ecotypes show high grazing tolerance, persistence, cold tolerance, and favorable forage production and quality [5].

Two major cytotypes occur in *P. notatum*: sexual diploids (2*n* = 2*x* = 20) and predominantly apomictic tetraploids (2*n* = 4*x* = 40) [6,7]. The association between polyploidy and apomixis is widespread in *Paspalum* [8]. Gametophytic apomixis comprises three developmental components: apomeiosis, parthenogenesis, and endosperm development. In *P. notatum*, apomeiosis occurs through apospory, in which a nucellar cell differentiates and develops into an unreduced embryo sac [9,10]. Because apospory represents only the formation of the unreduced female gametophyte, cytoembryological detection of aposporous embryo sacs does not, by itself, demonstrate parthenogenesis or clonal seed formation [11,12].

Genetic studies on *P. notatum* support the association of apospory with a major dominant factor located within the Apomixis Controlling Locus (ACL), a genomic region characterized by strongly restricted recombination [13,14]. In autotetraploid backgrounds, polysomic inheritance, preferential chromosome pairing near the ACL, segregation distortion, and incomplete penetrance can complicate simple Mendelian expectations [13,14]. More recent work has refined the molecular framework of apomixis in *Paspalum*, identifying a large, structurally complex apomixis-associated region and candidate regulatory pathways involved in apospory, including TGS1- and QGJ-related mechanisms [15,16]. At the breeding-population level, Marcón et al. [17] recently showed that segregation distortion and variable apospory expressivity remain important features of *P. notatum* progenies, reinforcing the need to evaluate inheritance and expressivity jointly.

In this context, the objective of this study was to determine the reproductive phenotype at the embryo-sac level in intraspecific tetraploid hybrids of *P. notatum*, quantify apospory expressivity in facultatively aposporous individuals, evaluate segregation at both family and pooled-population levels, and identify hybrids of interest for subsequent stages of the breeding program. We hypothesized that the progenies would segregate for the presence of apospory and that apospory expressivity would vary among hybrids.

## 2. Materials and Methods

Controlled crosses were performed during the summer of 2021 in a greenhouse at the Department of Forage Plants, Federal University of Rio Grande do Sul (UFRGS), Porto Alegre, Brazil. Female flowers at anthesis were emasculated and manually pollinated with fresh pollen collected at anthesis, following protocols adapted from Acuña et al. [18] and Weiler et al. [19]. Fertilized inflorescences were bagged, harvested approximately 30 days after pollination for seed extraction, and the resulting seeds were sown.

A total of 109 hybrids from 15 crosses were established under field conditions at the Agronomic Experimental Station of UFRGS (EEA), Eldorado do Sul, RS, Brazil (BR-290, km 146), in spring 2022. Inflorescences were successfully collected from 84 hybrids representing 11 crosses (Table 1). The remaining 25 hybrids either did not produce inflorescences during the sampling period or died before reproductive evaluation. The four unevaluated families shared the same male parent, PN17P3. Because reproductive phenotype could not be determined in plants that died or failed to flower, the analyzed subset may not fully represent the original 109-hybrid population, and a possible association between survival or flowering ability and reproductive phenotype cannot be excluded.

Both parents used in each cross were known tetraploids (2*n* = 4*x* = 40). Chromosome number was not directly determined in the F1 hybrids; therefore, tetraploidy of the progenies was expected from parental ploidy but was not cytologically confirmed in this study. The reproductive phenotypes of KF4, KD7, KD9, KF6, KF17, and KM2 had previously been determined by cytoembryological analysis [20]. The reproductive phenotype of the PN-series parents was also determined by cytoembryological analysis of cleared ovaries within the breeding program and is presented in Table 1. Crosses used sexual plants as female parents and aposporous plants as pollen donors.

The hybrids belong to an ongoing forage-breeding program and are also being evaluated for agronomic traits. Sexual genotypes can be used in subsequent crossing cycles, whereas hybrids with high apospory expressivity are candidates for additional reproductive and agronomic validation. Because the present study assessed embryo-sac development only, confirmation of clonal seed reproduction in candidate hybrids requires complementary evidence of parthenogenesis and maternal progeny origin.

For reproductive analysis, inflorescences at anthesis were collected between January and March 2023 and fixed in FAA solution (40 mL 95% ethanol, 14 mL distilled water, 3 mL 40% formalin, and 3 mL glacial acetic acid) for 24 h at room temperature. Samples were subsequently stored in 70% ethanol under refrigeration. Ovaries were dissected under a stereomicroscope, cleared through an alcohol series and methyl salicylate, mounted on microscope slides, and examined by differential interference contrast microscopy. Between 30 and 50 ovaries were evaluated per hybrid.

Ovules containing only a meiotic *Polygonum*-type embryo sac with antipodal cells were classified as sexual (S), whereas an ovule was classified as aposporous (A) when at least one aposporous embryo sac lacking the typical antipodal-cell organization was observed, whether alone or together with a sexual embryo sac [10,21]. Ovules that could not be reliably classified were recorded as unidentified (UI). A hybrid was classified as sexual only when all classified ovules were sexual. A hybrid was classified as facultatively aposporous (FAp) when at least one classified ovule contained an aposporous embryo sac while sexual ovules were also present. Apospory expressivity was calculated as A/(S + A) × 100; UI ovules were excluded from the denominator. Hybrids for which reproductive phenotype could not be established were classified as indeterminate (I).

Segregation analyses included the 80 hybrids with a conclusive reproductive classification; four indeterminate hybrids were excluded. A 1:1 sexual:facultatively aposporous ratio was used as the primary reference expectation because such a ratio is compatible with transmission of a dominant apospory-associated allele from a simplex tetraploid parent (Aaaa) crossed with a nulliplex sexual parent (aaaa), assuming polysomic inheritance, random segregation, no double reduction, and no gametic or zygotic selection [13,14]. Allelic dosage was not determined in the parents used here; therefore, the 1:1 ratio was treated as a reference model rather than as a confirmed parental-genotype model. For families with at least five classified hybrids, two-sided exact binomial tests were used to test a 0.5 probability of each reproductive class. The pooled 32 S:48 FAp population was evaluated with Pearson’s chi-square goodness-of-fit test only as complementary evidence. Heterogeneity in reproductive-class frequencies among families was evaluated with a Fisher–Freeman–Halton exact test using 500,000 Monte Carlo replicates. Ratios of 1:3 and 3:1 reported in previous *Paspalum* studies were considered in the literature comparison but were not treated as primary segregation hypotheses for these crosses. Statistical analyses were conducted in R version 4.6.0 [22], with significance set at *p* < 0.05.

## 3. Results

### 3.1. Reproductive Mode

**Figure 1:**
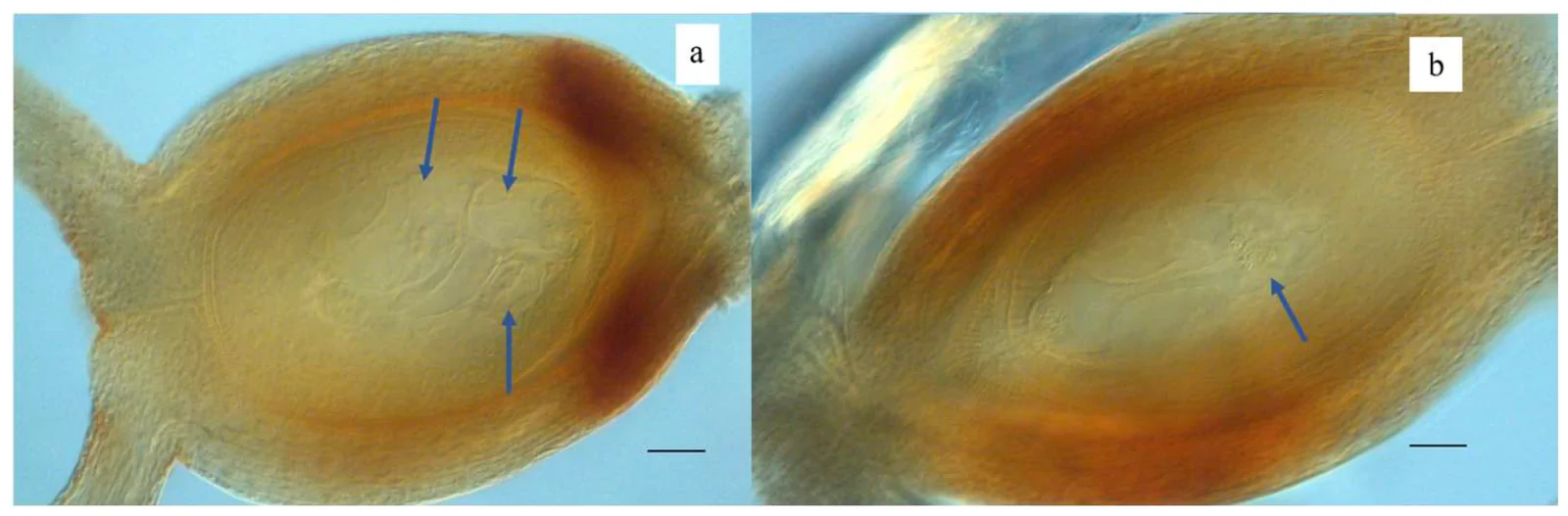
(**a**) Aposporous ovary showing multiple embryo sacs (arrows). (**b**) Sexual embryo sac with antipodal cells (arrow). Scale bar: 10 μm.

Among the 84 hybrids evaluated, reproductive phenotype was conclusively determined in 80 individuals. Based on the predefined criterion, 48 hybrids were classified as facultatively aposporous because at least one ovule contained an aposporous embryo sac and sexual ovules were also observed. The remaining 32 classified hybrids showed exclusively sexual ovules and were classified as sexual (Figure 1).

Four hybrids remained indeterminate because most ovaries were sterile, atrophied, aborted, or insufficiently developed for reliable embryo-sac classification (Table 2). In hybrids A20, A29, and E405, sexual and aposporous embryo sacs occurred within the same ovule, directly illustrating coexistence of the two developmental pathways.

**Figure 2:**
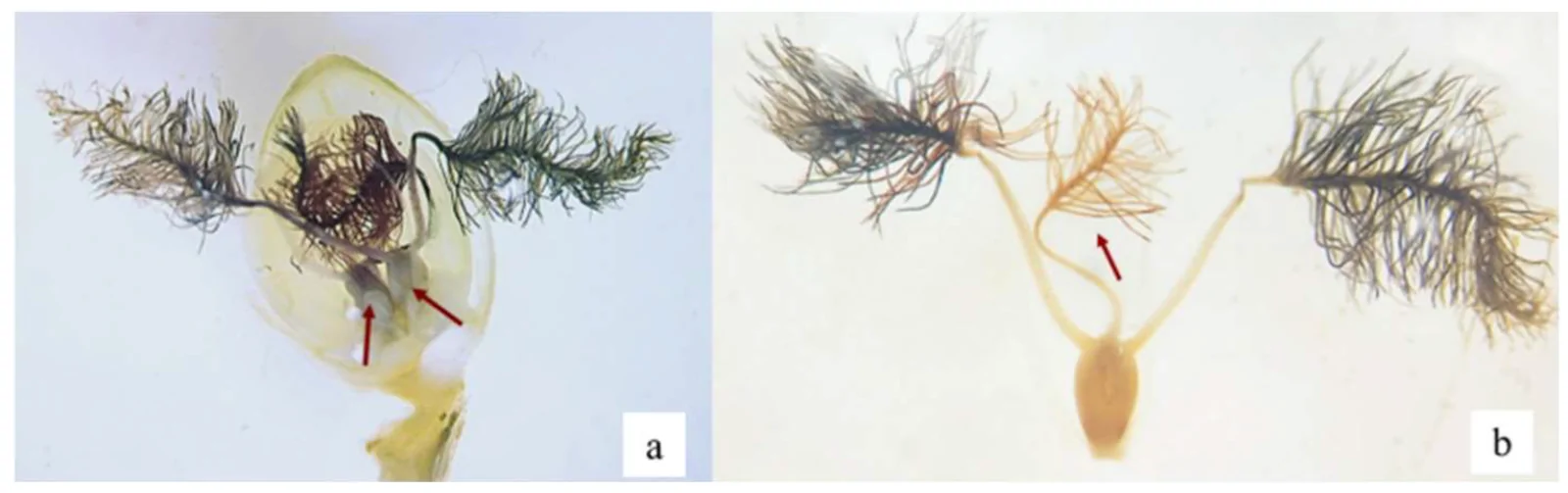
(**a**) Two complete pistils (arrows) within a single floral structure of hybrid A11; (**b**) Ovary of hybrid B104 with three stigmas; the arrow indicates the third stigma.

Additional floral abnormalities were recorded in two hybrids. Hybrid A11 showed two complete pistils within a single floral structure, whereas B104 showed three stigmas (Figure 2). These observations are reported as reproductive morphological variation; the present study was not designed to test an association between these abnormalities and apospory.

### 3.2. Apospory Expressivity

All 48 hybrids classified as aposporous also retained sexual ovules and were therefore classified as facultatively aposporous. Apospory expressivity varied markedly among individuals (Table 2), ranging from 3% to 69%.

The highest expressivity values occurred in A51 (69%), A29 (68%), A13 (59%), and A35 (56%), all from the KF4 × KF6 family. The lowest values occurred in A7, A21, A23, and D302 (3–5%).

Families A (KF4 × KF6) and B (KD9 × KF6) shared the same male parent, KF6, previously characterized as having low apospory expressivity (7%). Despite this common male parent, expressivity varied widely within and between the two families.

### 3.3. Apospory Segregation

Family sizes were highly unequal, ranging from 49 evaluated hybrids in KF4 × KF6 to one hybrid in several crosses (Table 3). Among families with at least five classified individuals, KF4 × KF6 deviated significantly from the 1:1 reference ratio (15 S:32 FAp; two-sided exact binomial *p* = 0.0186). KD9 × KF6 showed 6 S:6 FAp (*p* = 1.0000), KF17 × PN14P3 showed 1 S:4 FAp (*p* = 0.3750), and KM2 × KF6 showed 3 S:2 FAp (*p* = 1.0000).

The Fisher–Freeman–Halton exact test did not detect significant heterogeneity in reproductive-class frequencies among families (Monte Carlo *p* = 0.1444). At the pooled-population level, 32 sexual and 48 facultatively aposporous hybrids did not significantly deviate from a 1:1 ratio (χ^2^ = 3.20; df = 1; *p* = 0.0736). Because the families differed substantially in size and the largest family itself deviated from 1:1, the pooled result was interpreted only as complementary evidence rather than as a population-wide demonstration of Mendelian segregation.

## 4. Discussion

### 4.1. Reproductive Mode

The presence of apospory in 48 of the 80 classified hybrids confirms that the trait was transmitted in a substantial proportion of the progeny. However, all aposporous hybrids also retained sexual ovules, supporting their classification as facultatively aposporous rather than obligately aposporous. The occurrence of sexual and aposporous embryo sacs within the same ovule in A20, A29, and E405 further demonstrates that both developmental pathways can coexist within an individual.

Comparable coexistence of sexual and aposporous embryo sacs has been reported in *P. notatum* hybrids [20] and in tetraploid *Paspalum malacophyllum* [23]. In facultative aposporous grasses, the presence and relative development of the two embryo-sac pathways can vary among ovules and developmental stages. Importantly, cytoembryological observation establishes the presence of apospory but does not demonstrate the subsequent parthenogenetic development of the embryo or maternal origin of the progeny. Therefore, the present results should not be interpreted as direct confirmation of complete apomictic seed reproduction.

The double-pistil phenotype in A11 and the three-stigma phenotype in B104 indicate additional morphological variation during floral development. Because these abnormalities were observed incidentally and were not tested experimentally for association with reproductive phenotype, no causal relationship with apospory is inferred here.

### 4.2. Apospory Expressivity

Apospory expressivity varied from 3% to 69%, demonstrating substantial quantitative variation among facultatively aposporous hybrids. This broad range is consistent with previous studies showing strong genotype-dependent variation in apospory expression in *P. notatum* and related *Paspalum* breeding populations [17,24,25,26,27].

The variation observed here is relevant to breeding because apospory expressivity provides information on the frequency with which unreduced embryo sacs are formed. Machado et al. [24], Zilli et al. [26], and Marcón et al. [17] likewise reported considerable variation among *P. notatum* hybrids, while Brugnoli et al. [27] demonstrated broad reproductive variation in novel *Paspalum* hybrids. Together, these studies indicate that the presence of apospory and its level of expression should be evaluated separately.

Variation in apospory expressivity is compatible with incomplete penetrance and with additional genetic, epigenetic, developmental, and environmental modulation of the trait [15,16,26]. The molecular architecture of the ACL and the regulatory networks associated with apospory are complex, and the present study did not genotype the ACL or determine allelic dosage. Consequently, the observed variation cannot be attributed to a specific molecular mechanism from the current data.

From a breeding perspective, high apospory expressivity can be used to prioritize hybrids for further reproductive evaluation, but it should not be treated as evidence that a genotype will necessarily reproduce clonally through seed. Complete apomixis also requires parthenogenesis and appropriate endosperm development, which were not evaluated here [11,12]. Thus, progeny testing, molecular verification of maternal origin, or seed-based cytometric approaches are needed before clonal seed reproduction can be confirmed.

Within this framework, A51, A29, A13, and A35 are promising candidates for subsequent breeding stages because they combined the highest apospory expressivity values in the evaluated population. Their value as potential cultivar candidates, however, will depend on agronomic performance and on direct confirmation of complete apomictic seed reproduction.

### 4.3. Apospory Segregation

The revised family-level analysis changes the interpretation of segregation. Although the pooled 32 S:48 FAp distribution did not significantly deviate from 1:1, this result was strongly influenced by the unequal contribution of families. The largest family, KF4 × KF6, showed a significant excess of facultatively aposporous hybrids relative to the 1:1 reference (15:32; exact *p* = 0.0186), whereas the other adequately sized families did not depart from 1:1. The overall exact test of heterogeneity among families was not significant, but statistical power was limited by the very small size of most families.

Previous studies on *P. notatum* have frequently reported segregation distortion in the opposite direction, with an excess of sexual individuals. Martínez et al. [13] and Stein et al. [14] described strong distortion against apospory, while Zilli et al. [26] reported family-dependent ratios ranging from approximately 1:1 to markedly sexuality-biased segregation. Marcón et al. [17] recently observed sexual:apomictic ratios of 5:1 and 4.3:1 in progenies derived from two recurrent-selection methods. In contrast, Krycki et al. [20] reported a ratio close to 1:1 in a smaller intraspecific population.

Family-dependent segregation has also been observed in other breeding populations. Acuña et al. [28] reported distorted segregation in tetraploid bahiagrass hybrids, and interspecific *Paspalum* crosses have produced ratios that also depend on parental combination and genetic background [29,30]. These findings emphasize that a pooled ratio can mask biologically relevant family-level behavior.

Segregation distortion in *Paspalum* has been associated with the genomic region controlling apospory and with reduced transmission or viability of gametes or zygotes carrying the apospory-associated factor [13,14,15]. In the present material, however, the largest family showed an excess rather than a deficit of aposporous individuals. One possible explanation is that the parental genetic backgrounds used here did not impose the viability constraints previously associated with the apomixis-controlling region; however, this interpretation remains speculative and requires molecular validation.

The current data do not establish the allelic dosage of the apospory-associated factor in any parent. Therefore, although a 1:1 ratio is compatible with a simplex dominant model under specific autotetraploid assumptions, neither simplex dosage nor a single-locus Mendelian mechanism can be demonstrated from the segregation data alone. Potential effects of double reduction, preferential chromosome pairing, linked viability factors, and incomplete penetrance should also be considered when interpreting tetraploid segregation [13,14,15].

Taken together, the results indicate that the inheritance of the presence of apospory and the quantitative expression of apospory should be treated as related but distinct features. Molecular and epigenetic studies on *P. notatum* support a complex regulatory architecture superimposed on the major apospory-associated region [15,16], providing a plausible basis for the wide expressivity observed among hybrids.

For breeding, these findings reinforce the value of evaluating reproductive phenotype at the family and individual levels. Sexual hybrids remain useful for recombination and recurrent crossing, whereas highly aposporous hybrids can be advanced for agronomic testing and for direct confirmation of clonal seed reproduction.

The unequal family sizes and the absence of reproductive data for hybrids that died or failed to flower are important limitations of the present study. Future evaluations with more balanced family sizes, direct ploidy confirmation, molecular characterization of the apomixis-associated region, and progeny-based assessment of parthenogenesis would allow stronger genetic inference and a more complete characterization of reproductive behavior.

## 5. Conclusions

Cytoembryological analysis classified 48 hybrids as facultatively aposporous and 32 as sexual, while four remained indeterminate. Apospory expressivity ranged from 3% to 69%, identifying A51, A29, A13, and A35 as candidates for further evaluation. The pooled population did not significantly deviate from a 1:1 sexual:facultatively aposporous ratio, but the largest family showed a significant excess of aposporous hybrids, demonstrating why family-level analysis is necessary in progenies of unequal size. Overall heterogeneity among families was not significant. Because parthenogenesis and maternal progeny origin were not evaluated, the conclusions are restricted to the presence and expressivity of apospory rather than complete apomictic seed reproduction.

## Figures and Tables

**Table 1:** Crosses, parental reproductive phenotype, number of hybrids evaluated per cross, and hybrid identification in *P. notatum*.

Crosses				Hybrids	
♀ (2*n* = 4*x* = 40) *		♂ (2*n* = 4*x* = 40),		No. of Hybrids	Hybrid ID
KF4	S **	KF6	FA ***	49	A1-A59
KD9	S	KF6	FAp	12	B101-B113
KF17	S	PN14P3	FAp	5	C201-C207
KM2	S	KF6	FAp	5	D301-D307
KF17	S	KD7	FAp	4	E401-E405
KM2	S	PN2P6	FAp	2	F501-F502
KF17	S	PN10P3	FAp	2	G601-G602
KM2	S	PN3P3	FAp	1	H702
KF17	S	PN9P3	FAp	2	I801-I802
KF4	S	PN1P2	FAp	1	J1102
KD9	S	PN5P1	FAp	1	K1201

Note: *: 2*n* = 4*x* = 40 refers to the parental genotypes. F1 chromosome number was not directly determined. **: S = sexual; ***: FAp = facultatively aposporous based on cytoembryological analysis.

**Table 2:** Reproductive classification and apospory expressivity of F1 hybrids of *P. notatum*.

Plant ID	Female Parent	Male Parent	S	A	UI	T	Reprod. Class	Apospory (%)
A1	KF 4	KF 6	29	2	9	40	FAp	6%
A2	KF 4	KF 6	31	11	8	50	FAp	26%
A3	KF 4	KF 6	36	5	5	46	FAp	12%
A4	KF 4	KF 6	25	14	6	45	FAp	36%
A5	KF 4	KF 6	29	4	4	37	FAp	12%
A6	KF 4	KF 6	33	0	9	42	S	-
A7	KF 4	KF 6	32	1	2	35	FAp	3%
A8	KF 4	KF 6	21	11	7	39	FAp	34%
A9	KF 4	KF 6	28	8	3	39	FAp	22%
A11	KF 4	KF 6	32	2	4	38	FAp	6%
A12	KF 4	KF 6	34	0	11	45	S	-
A13	KF 4	KF 6	15	22	4	41	FAp	59%
A15	KF 4	KF 6	18	14	10	42	FAp	44%
A16	KF 4	KF 6	29	0	9	38	S	-
A17	KF 4	KF 6	30	0	6	36	S	-
A18	KF 4	KF 6	29	5	5	39	FAp	15%
A19	KF 4	KF 6	34	0	0	34	S	-
A20	KF 4	KF 6	33	4	2	39	FAp	11%
A21	KF 4	KF 6	34	1	8	43	FAp	3%
A22	KF 4	KF 6	35	4	5	44	FAp	10%
A23	KF 4	KF 6	35	1	8	44	FAp	3%
A26	KF 4	KF 6	23	17	5	45	FAp	43%
A27	KF 4	KF 6	UI	UI	UI	UI	I	-
A28	KF 4	KF 6	36	3	3	42	FAp	8%
A29	KF 4	KF 6	10	21	12	43	FAp	68%
A30	KF 4	KF 6	27	8	4	39	FAp	23%
A31	KF 4	KF 6	30	0	8	38	S	-
A32	KF 4	KF 6	32	0	2	34	S	-
A34	KF 4	KF 6	34	0	4	38	S	-
A35	KF 4	KF 6	16	20	2	38	FAp	56%
A36	KF 4	KF 6	35	0	12	47	S	-
A37	KF 4	KF 6	31	2	6	39	FAp	6%
A38	KF 4	KF 6	30	5	5	40	FAp	14%
A41	KF 4	KF 6	36	0	10	46	S	-
A42	KF 4	KF 6	31	5	8	44	FAp	14%
A43	KF 4	KF 6	37	0	8	45	S	-
A44	KF 4	KF 6	33	3	2	38	FAp	8%
A45	KF 4	KF 6	UI	UI	UI	UI	I	-
A46	KF 4	KF 6	33	0	4	37	S	-
A48	KF 4	KF 6	33	2	3	38	FAp	6%
A49	KF 4	KF 6	26	12	6	44	FAp	32%
A50	KF 4	KF 6	31	0	4	35	S	-
A51	KF 4	KF 6	10	22	12	44	FAp	69%
A52	KF 4	KF 6	34	0	6	40	S	-
A53	KF 4	KF 6	31	7	7	45	FAp	18%
A54	KF 4	KF 6	36	0	0	36	S	-
A55	KF 4	KF 6	20	12	15	47	FAp	38%
A56	KF 4	KF 6	35	4	2	41	FAp	10%
A57	KF 4	KF 6	31	2	0	33	FAp	6%
B101	KD9	KF6	34	5	5	44	FAp	13%
B102	KD9	KF6	30	2	2	34	FAp	6%
B103	KD9	KF6	32	0	12	44	S	-
B104	KD9	KF6	31	0	16	47	S	-
B105	KD9	KF6	34	3	2	39	FAp	8%
B106	KD9	KF6	34	15	1	50	FAp	31%
B107	KD9	KF6	31	0	3	34	S	-
B108	KD9	KF6	30	0	4	34	S	-
B110	KD9	KF6	32	2	11	45	FAp	6%
B111	KD9	KF6	31	0	4	35	S	-
B112	KD9	KF6	32	0	7	39	S	-
B113	KD9	KF6	33	5	4	42	FAp	13%
C202	KF17	PN14P3	19	22	8	49	FAp	54%
C203	KF17	PN14P3	31	5	5	41	FAp	14%
C204	KF17	PN14P3	31	0	19	50	S	-
C205	KF17	PN14P3	25	7	15	47	FAp	22%
C207	KF17	PN14P3	30	10	2	42	FAp	25%
D301	KM2	KF6	32	0	13	45	S	-
D302	KM2	KF6	35	2	4	41	FAp	5%
D304	KM2	KF6	32	0	7	39	S	-
D306	KM2	KF6	26	6	10	42	FAp	19%
D307	KM2	KF6	35	0	5	40	S	-
E401	KF17	KD7	27	11	4	42	FAp	29%
E402	KF17	KD7	32	0	12	44	S	-
E403	KF17	KD7	33	0	5	38	S	-
E405	KF17	KD7	32	9	5	46	FAp	22%
F501	KM2	PN2P6	31	0	11	42	S	-
F502	KM2	PN2P6	32	0	13	45	S	-
G601	KF17	PN10P3	32	0	6	38	S	-
G602	KF17	PN10P3	35	0	12	47	S	-
H702	KM2	PN3P3	34	7	3	44	FAp	17%
I801	KF17	PN9P3	31	0	15	46	S	-
I802	KF17	PN9P3	UI	UI	UI	UI	I	-
J1102	KF4	PN1P2	36	6	0	42	FAp	14%
K1201	KD9	PN5P1	UI	UI	UI	UI	I	-

S = sexual ovules; A = ovules containing at least one aposporous embryo sac; UI = unidentified ovules; T = total number of ovaries analyzed. Reproductive classification: S = sexual; FAp = facultatively aposporous; I = indeterminate. Apospory expressivity = A/(S + A) × 100.

**Table 3:** Family-level segregation of sexual and facultatively aposporous F1 hybrids of *P. notatum*.

Cross	Evaluated *n*	S	FAp	I	S:FAp	*p*-Value vs. 1:1
KF4 × KF6	49	15	32	2	15:32	0.0186
KD9 × KF6	12	6	6	0	6:6	1.0000
KF17 × PN14P3	5	1	4	0	1:4	0.3750
KM2 × KF6	5	3	2	0	3:2	1.0000
KF17 × KD7	4	2	2	0	2:2	—
KM2 × PN2P6	2	2	0	0	2:0	—
KF17 × PN10P3	2	2	0	0	2:0	—
KM2 × PN3P3	1	0	1	0	0:1	—
KF17 × PN9P3	2	1	0	1	1:0	—
KF4 × PN1P2	1	0	1	0	0:1	—
KD9 × PN5P1	1	0	0	1	—	—
Pooled population	84	32	48	4	32:48	0.0736

FAp = facultatively aposporous; I = indeterminate. Family-level *p* values are two-sided exact binomial tests against a 1:1 ratio and are reported for families with at least five classified hybrids. The pooled p value is from Pearson’s chi-square goodness-of-fit test and is presented only as complementary evidence. Across informative families, the Fisher–Freeman–Halton exact test with 500,000 Monte Carlo replicates indicated no significant heterogeneity (*p* = 0.1444).

## Data Availability

The data supporting the findings of this study are included in the article. Further inquiries can be directed to the corresponding authors.

## Abbreviations

The following abbreviations are used in this manuscript:

ACL	Apomixis Controlling Locus
FAp	facultatively aposporous
S	sexual
UI	unidentified

## References

[1] Overbeck G.E., Vélez-Martin E., Menezes L.S., Anand M., Baeza S., Carlucci M.B., Dechoum M.S., Durigan G., Fidelis A., Guido A. (2022). Placing Brazil’s grasslands and savannas on the map of science and conservation. Perspect. Plant Ecol. Evol. Syst..

[2] Wróbel B., Zielewicz W., Paszkiewicz-Jasińska A. (2025). Improving forage quality from permanent grasslands to enhance ruminant productivity. Agriculture.

[3] Zuloaga F.O., Morrone O. (2005). Revisión de las Especies de Paspalum para América del Sur Austral (Argentina, Bolivia, Sur del Brasil, Chile, Paraguay y Uruguay).

[4] Steiner M.G., Dall’Agnol M., Nabinger C., Scheffer-Basso S.M., Weiler R.L., Simioni C., Schifino-Wittmann M.T., da Motta E.A.M. (2017). Forage potential of native ecotypes of *Paspalum notatum* and *P. guenoarum*. An. Acad. Bras. Cienc..

[5] Souza F.H.D., Cavallari M.M., Gusmão M.F. (2020). Produção Comercial de Sementes de Paspalum notatum var. notatum.

[6] Burton G.W. (1955). Breeding Pensacola bahiagrass, *Paspalum notatum*: I. Method of reproduction. Agron. J..

[7] Burton G.W. (1948). The method of reproduction in common bahiagrass, *Paspalum notatum*. J. Am. Soc. Agron..

[8] Quarín C.L., Norrmann G.A. (1990). Cytology and reproductive behavior of Paspalum species. Plant Syst. Evol..

[9] Nogler G.A., Johri B.M. (1984). Gametophytic apomixis. Embryology of Angiosperms.

[10] Quarín C.L., Espinoza F., Martínez E.J., Pessino S.C., Bovo O.A. (2001). A rise of ploidy level induces the expression of apomixis in *Paspalum notatum*. Sex. Plant Reprod..

[11] Hojsgaard D. (2020). Apomixis technology: Separating the wheat from the chaff. Genes.

[12] Xu Y., Jia H., Tan C., Wu X., Deng X., Xu Q. (2022). Apomixis: Genetic basis and controlling genes. Hortic. Res..

[13] Martínez E.J., Urbani M.H., Quarin C.L., Ortiz J.P.A. (2001). Inheritance of apospory in bahiagrass, *Paspalum notatum*. Hereditas.

[14] Stein J., Quarin C.L., Martínez E.J., Pessino S.C., Ortiz J.P.A. (2004). Tetraploid races of *Paspalum notatum* show polysomic inheritance and preferential chromosome pairing around the apospory-controlling locus. Theor. Appl. Genet..

[15] Ortiz J.P.A., Pupilli F., Acuña C.A., Leblanc O., Pessino S.C. (2020). How to become an apomixis model: The multifaceted case of *Paspalum*. Genes.

[16] Colono C.M., Podio M., Siena L.A., Ortiz J.P.A., Leblanc O., Pessino S.C. (2022). Spotting the targets of the apospory controller TGS1 in *Paspalum notatum*. Plants.

[17] Marcón F., Lezcano Galanter I.N., Brugnoli E.A., Zilli A.L., Martínez E.J., Acuña C.A. (2024). Comparing two recurrent selection methods for developing superior apomictic hybrids in *Paspalum notatum*. Crop Sci..

[18] Acuña C.A., Blount A.R., Quesenberry K.H., Hanna W.W., Kenworthy K.E. (2007). Reproductive characterization of bahiagrass germplasm. Crop Sci..

[19] Weiler R.L., Dall’Agnol M., Simioni C., Krycki K.C., Dahmer N., Guerra D. (2017). Determination of the mode of reproduction of bahiagrass hybrids using cytoembryological analysis and molecular markers. Rev. Bras. Zootec..

[20] Krycki K.C., Tedesco M., Rodrigues V., Weiler R.L., Brunes A.P., Dall’Agnol M., Simioni C. (2020). Reproductive analyses of intraspecific *Paspalum notatum* Flügge hybrids. Crop Breed. Appl. Biotechnol..

[21] Espinoza F., Urbani M.H., Martínez E.J., Quarin C.L. (2001). The breeding system of three *Paspalum* species with forage potential. Trop. Grassl..

[22] R Core Team (2026). R: A Language and Environment for Statistical Computing.

[23] Hojsgaard D.H., Martínez E.J., Quarin C.L. (2013). Competition between meiotic and apomictic pathways during ovule and seed development results in clonality. New Phytol..

[24] Machado J.M., Krycki K.C., Weiler R.L., Simioni C., Dall’Agnol M. (2021). Reproduction mode and apospory expressivity of selected hybrids of *Paspalum notatum* Flügge. J. Plant Breed. Crop Sci..

[25] Stein J., Pessino S.C., Martínez E.J., Rodriguez M.P., Siena L.A., Quarin C.L., Ortiz J.P.A. (2007). A genetic map of tetraploid *Paspalum notatum* Flügge (bahiagrass) based on single-dose molecular markers. Mol. Breed..

[26] Zilli A.L., Brugnoli E.A., Marcón F., Billa M.B., Rios E.F., Martínez E.J., Acuña C.A. (2015). Heterosis and expressivity of apospory in tetraploid bahiagrass hybrids. Crop Sci..

[27] Brugnoli E.A., Zilli A.L., Marcón F., Caballero E., Martínez E.J., Acuña C.A. (2023). Reproductive and agronomic characterization of novel apomictic hybrids of *Paspalum* (Poaceae). Genes.

[28] Acuña C.A., Blount A.R., Quesenberry K.H., Kenworthy K.E., Hanna W.W. (2011). Tetraploid bahiagrass hybrids: Breeding technique, genetic variability and proportion of heterotic hybrids. Euphytica.

[29] Aguilera P.M., Galdeano F., Quarin C.L., Ortiz J.P.A., Espinoza F. (2015). Inheritance of aposporous apomixis in interspecific hybrids derived from sexual *Paspalum plicatulum* and apomictic *Paspalum guenoarum*. Crop Sci..

[30] Aguilera P.M., Sartor M.E., Galdeano F., Espinoza F., Quarin C.L. (2011). Interspecific tetraploid hybrids between two forage grass species: Sexual *Paspalum plicatulum* and apomictic *P. guenoarum*. Crop Sci..

